# Effects of Dietary l-Glutamine Supplementation on the Intestinal Function and Muscle Growth of Piglets

**DOI:** 10.3390/life14030405

**Published:** 2024-03-19

**Authors:** Lei Wang, Meng Shen, Jiale Liu, Yanyan Zhang, Zhekun Zhu, Baocheng Li, Shuangshuang Guo, Dan Yi, Binying Ding, Tao Wu, Di Zhao, Kang Yao, Yongqing Hou

**Affiliations:** 1 Key Laboratory of Agro-Ecological Processes in Subtropical Region, Institute of Subtropical Agriculture, Chinese Academy of Sciences, Changsha 410125, China; wanglei@whpu.edu.cn; 2 University of Chinese Academy of Sciences, Beijing 100039, China; 3 Hubei Collaborative Innovation Center for Animal Nutrition and Feed Safety, Hubei Key Laboratory of Animal Nutrition and Feed Science, Wuhan Polytechnic University, Wuhan 430023, China; 15202740663@163.com (M.S.); 17303861138@163.com (J.L.); zhangyanyan@whpu.edu.cn (Y.Z.); zzk961207@outlook.com (Z.Z.); baocheng2023@126.com (B.L.); guo1shuangshuang@163.com (S.G.); yidan810204@whpu.edu.cn (D.Y.); dbying7471@126.com (B.D.); wutao@whpu.edu.cn (T.W.); zhaodi@whpu.edu.cn (D.Z.)

**Keywords:** piglets, glutamine, intestinal function, longissimus dorsi muscle, muscle growth

## Abstract

The aim of this study was to investigate the effects of dietary l-glutamine (Gln) supplementation on the morphology and function of the intestine and the growth of muscle in piglets. In this study, sixteen 21-day-old piglets were randomly divided into two groups: the Control group (fed a basal diet) and the Gln group (fed a basal diet supplemented with 0.81% Gln). Blood, gut, and muscle samples were collected from all piglets on Day 20 of the trial. Compared with the Control group, the supplementation of Gln increased (*p* < 0.05) the villus height, villus width, villus surface area, and villus height/crypt depth ratio of the small intestine. Furthermore, the supplementation of Gln increased (*p* < 0.05) total protein, total protein/DNA, and RNA/DNA in both the jejunum and ileum. It also increased (*p* < 0.05) the concentrations of carnosine and citrulline in the jejunal mucosa, as well as citrulline and cysteine concentrations in the ileum. Conversely, Gln supplementation decreased (*p* < 0.05) Gln concentrations in both the jejunum and ileum, along with β-aminoisobutyric acid and 1-Methylhistidine concentrations, specifically in the ileum. Subsequent research revealed that Gln supplementation increased (*p* < 0.05) the mRNA levels for glutathione-S-transferase omega 2 and interferon-*β* in the duodenum. In addition, Gln supplementation led to an increase (*p* < 0.05) in the number of *Lactobacillus* genus in the colon, but a decrease (*p* < 0.05) in the level of HSP70 in the jejunum and the activity of diamine oxidase in plasma. Also, Gln supplementation reduced (*p* < 0.05) the mRNA levels of glutathione-S-transferase omega 2 and interferon stimulated genes, such as *MX1*, *OAS1*, *IFIT1*, *IFIT2*, *IFIT3*, and *IFIT5* in both the jejunum and ileum, and the numbers of *Clostridium coccoides*, *Enterococcus* genus, and *Enterobacterium* family in the colon. Moreover, Gln supplementation enhanced (*p* < 0.05) the concentrations of total protein, RNA/DNA, and total protein/DNA ratio in the longissimus dorsi muscle, the concentrations of citrulline, ornithine, arginine, and hydroxyproline, and the mRNA level of peptide transporter 1, while reducing the contents of hydrogen peroxide and malondialdehyde and the mRNA level of glutathione-S-transferase omega 2 in the longissimus dorsi muscle. In conclusion, dietary Gln supplementation can improve the intestinal function of piglets and promote the growth of the longissimus dorsi muscle.

## 1. Introduction

Piglets are subjected to continuous nutritional, psychological, and environmental stress during weaning, adversely affecting future growth performance, which can lead to significant economic losses in the pig industry. It is important to develop strategies which reduce the negative effects of stress after weaning. Gln is regarded as the most abundant free amino acid in the body. It is mainly synthesised in skeletal muscle which is the most important site for Gln storage [1]. Gln provides nitrogen and carbon skeletons for endogenous arginine synthesis [2], stimulates the mammalian target of rapamycin (mTOR) pathway [3], takes part in the synthesis of glutathione [4], and acts as a precursor for the synthesis of purine and pyrimidine nucleotides. In addition, Gln is involved in maintaining the integrity and function of the intestinal barrier and promoting cell proliferation [5], and is essential for the optimal growth of newborn piglets.

Numerous studies have shown that Gln can regulate the intestinal function of piglets [6,7,8]. As we know, the small intestine is the central place to absorb Gln [9,10,11], Gln not only promotes intestinal growth and development [12,13], but also increases the expression of anti-oxidation-related genes in the intestine [14]. In addition, Gln can regulate the gut microbiota [6,15,16], ultimately promoting the performance of weaned pigs. In this study, the expression levels of genes related to neutralizing glutamine metabolism in the duodenum, jejunum, ileum, and longissimus were analysed using Quantitative Real-Time PCR, verifying the direct regulatory effect of glutamine on intestinal and musculature-related genes. Amino acid concentrations in the jejunum, ileum, and longissimus tuneus were detected using an amino acid analyser. The obtained data can directly reflect the changes of various amino acid concentrations after adding glutamine. Finally, the combination of these two aspects can verify the beneficial effect of glutamine on intestinal health and muscle growth and provide a reference for its application.

## 2. Materials and Methods

### 2.1. Animal and Experimental Design

The experiment was approved by the Institutional Animal Care and Use Committee of Hubei Province (Approval Code WPU201809001). In this experiment, all pigs were born naturally at term (114 days of gestation). Sixteen 21 day-old healthy piglets (Duroc × Landrace × Yorkshire, 5.49 ± 0.35 kg) were purchased from commercial pig farms. All piglets were kept in individual cages and the ambient temperature was maintained at 22–25 °C [17]. Each cage was equipped with a plastic trough floor, a nipple water bottle, and a feeder to allow the pigs to feed and drink freely. According to the requirement of the National Research Council in 2012, the diet formula consists of corn and soybean meal, with specific additions, as shown in Table 1. After a 3-day adaptation period, piglets were randomly divided into two groups: the Control group and the Gln group. Each group contained 8 replicates with 1 pig per replicate. The experiment lasted for 20 days. Piglets in the Control group were fed a basal diet containing 0.99% l-alanine for isonitrogenous control, and the experimental group were fed a basal diet supplemented with 0.81% Gln.

### 2.2. Sample Collection

At the end of the experiment, blood samples were collected from the anterior vena cava of the piglets using 10 mL heparinised vacuum tubes after 12 h fasting, and it was placed at 37 °C for 30 min. The plasma was then separated by centrifuging at 3500× *g* for 10 min, and then stored at −80 °C until analysis. Piglets were then euthanised with an intravenous injection of pentobarbital sodium [18] (50 mg/kg BW). As described by Yi et al. [17], 1 cm intestinal segments of the descending duodenum, middle jejunum, and ileum were taken and placed in 4% paraformaldehyde for the preparation of gastrointestinal tract sections. Then, an approximately 10 cm long segment of the intestine was opened lengthwise and carefully rinsed with cold phosphate-buffered saline, followed by the scraping of the mucosa. Intestinal mucosal samples were wrapped in tinfoil, frozen with liquid nitrogen at −80 °C, and stored until use. At the same time, the longissimus dorsi muscle was collected from the left caudal side of the carcass to the tenth rib with a 3 cm long section and then stored at −80 °C for further experiments. Moreover, colonic chyme was collected into a 2 mL EP tube with a small hole, totalling 5 tubes of colonic chyme per piglet. These EP tubes were frozen in liquid nitrogen and stored for later use.

### 2.3. Intestinal Morphology Measurement

The intestine was dehydrated and paraffin-embedded [12]. The intestinal morphology was determined after 5 μm sections were stained with haematoxylin and eosin (H.E.) [19]. The intestinal villus morphology was measured using light microscopy (American Optical Co., Scientific Instrument Div., Buffalo, NY, USA) with a computer-assisted morphometry system (BioScan Optimetric, BioScan Inc., Edmonds, WA, USA). Villus height (VH) is the vertical distance from the tip of the villi to the crypt; crypt depth (CD) is the vertical distance from the crypt to the base; villus width (VW) is measured using an auxiliary linear measurement tool to take average values of uniform parts in the whole villi; and villus surface area (VS) is measured using the trace point of the regular polygon measuring tool in the auxiliary measuring system. The results of these indicators were determined by the average of ten villi from eight samples in each group.

### 2.4. Determination of DAO Activity in Plasma

According to Hampson et al. [20], the activity of diamine oxidase (DAO) in plasma was determined using spectrophotometry. The assay mixture (3.8 mL) contained 3 mL of phosphate buffer (0.2 M, pH 7.2), 0.1 mL (0.004%) of horseradish peroxidase solution (Sigma Chemicals, St. Louis, MI, USA), 0.1 mL of o-dianisidine–methanol solution [0.5% of o-dianisidine (Sigma Chemicals) in methanol], 0.5 mL of plasma, and 0.1 mL of substrate solution (0.175% of cadaverine dihydrochloride, Sigma Chemicals). The mixture was incubated at 37 °C for 30 min, and absorbance at 436 nm was measured to calculate the activity of DAO [21].

### 2.5. Assay of DNA, RNA, and Protein Contents in Intestinal Mucosa and Longissimus Dorsi Muscle

According to Hou et al. [22], the DNA, RNA, and protein contents were extracted from the mucosa and longissimus dorsi muscle using the TRI REAGENT-RNA/DNA/Protein isolation reagent (Nanjing Jiancheng Bioengineering Institute, Nanjing, China). The modified Schmidt–Tannhauser method, described by Johnson and Chandller [23], was used to determine DNA and RNA using spectrophotometry. Mucosa and muscle samples (~0.1 g) were homogenised in 1 mL of ice-cold PBS-EDTA buffer (0.05 mol/L Na_3_PO_4_, 2.0 mol/L NaCl, 2.0 mmol/L EDTA, pH 7.4). The homogenate was centrifuged at 12,000× *g* at 4 °C for 10 min to obtain the supernatant for assays. The contents of mucosal and muscle proteins were analysed according to the method of Lowry et al. [24].

### 2.6. Determination of Amino Acid Contents in Intestinal Mucosa and Longissimus Dorsi Muscle

Amino acids in the jejunum, ileum, and longissimus dorsi muscle were determined using an automated amino acid analyser (S433D, Sykam GmbH, Eresing, Germany), as described by Xie et al. [25], with minor modifications. Briefly, 1 mL of homogenate was thoroughly mixed with 1 mL of salicylsulphonic acid (2%); after a 15 min ice bath, the supernatant was collected by means of centrifugation at 10,000× *g* at 4 °C for 15 min, and then the lithium hydroxide solution was added to the supernatant to adjust to pH 7.0. The mixture was filtered through a 0.22 µm filter membrane and used for the determination of amino acid content. The chromatographic system consisted of a Waters Breeze HPLC system (Waters Corporation, Milford, MA, USA), including 1525 binary HPLC pumps, a 2487 Dual-λ Absorbance Detector, 717 plus autosampler and Breeze system software (version 3.30 SPA), Waters XTerra MS C18 column (5 μm × 4.6 mm × 150 mm), mobile phase A (0.1 mol/L sodium acetate, pH 7.2), and mobile phase B (100% methanol).

### 2.7. Quantitative Real-Time PCR Analyses for Gene Expression

Total RNA was extracted from the intestinal mucosa and longissimus dorsi muscle using TRIzol Reagent (Invitrogen, Carlsbad, CA, USA), according to the method provided by the manufacturer. The integrity of RNA was verified using agarose gel electrophoresis. The purity and concentration of RNA were assessed through the spectrophotometric determination of the OD_260_/OD_280_ ratio. The OD_260_/OD_280_ ratio of all samples was above 1.8, which corresponds to 90–100% pure nucleic acid [26,27]. Moreover, total RNA was reverse-transcribed by using the PrimeScript^®^RT kit combined with gDNA Eraser (Takara, Dalian, China). The cDNA was stored at −20 °C until use. To amplify the cDNA fragments of the mucosa, longissimus dorsi muscle, and microbia, qPCR was performed by using primer pairs (Table 2 as previously described by Ott et al. [28]. The analysis for gene expression was performed on the Applied Biosystems 7500 Real-Time PCR System (Foster City, CA, USA) by using SYBR^®^ Premix Ex Taq™ (Takara, Dalian, China), according to the manufacturer’s instructions. The specificity of the qRT-PCR reactions was assessed by analysing the melting curves of the products and verifying the size of the amplicons [29]. To ensure the sensitivity and accuracy of the qPCR results, both mucosal and longissimus dorsi muscle samples were internally normalised using the average cycle threshold (Ct) of ribosomal protein L4 and glyceraldehyde-3-phosphate dehydrogenase [30]; to avoid any artifacts caused by variants in the target genes, the average Ct of 16S rDNA [31] was used as the reference for each sample for normalisation of microbial samples. The analysis of the results was performed using the 2^−ΔΔCt^ method [32].

### 2.8. Quantitative PCR Analyses for Colon Bacteria

As described by Castillo et al. [33], the microbial DNA was extracted and purified from colon chyme using the QIAamp DNA Stool Mini Kit (Qiagen, West Sussex, UK). In short, each sample of frozen digestive fluid (0.3–0.5 g) was thawed and homogenised in the InhibitEX buffer and then centrifuged to obtain the supernatant. Proteinase K was added to the supernatant, mixed sufficiently, and centrifuged to collect the supernatant. Then, 200 μL of ethanol (96–100%) was added to the supernatant (200 μL), and the DNA was purified using the QIAamp spin column. The total DNA was quantified using a NanoDrop^®^ ND-1000A UV-vis spectrophotometer (Thermo Scientific, Wilmington, DE, USA) at an OD value of 260 nm, and its purity was evaluated by measuring the OD_260_/OD_280_ ratio. The OD_260_/OD_280_ ratios of all samples were between 1.7 and 1.9. In addition, the genomic DNA length of each sample was determined using 1% denatured agarose gel electrophoresis. Samples were stored at −20 °C until qPCR analysis.

### 2.9. Protein Immunoblot Analysis

Western blotting was used to analyse the HSP70 protein, as described by Hou et al. [21,34]. In short, the frozen intestinal mucosa samples were ground into powder using a mortar and pestle under liquid nitrogen conditions. A jejunum mucosal sample of approximately 100 mg was taken and mixed with 1 mL lysis buffer, after which the homogeniser was used to configure the supernatant at 12,000× *g* at 4 °C for 15 min. The supernatant was added to microcentrifuge tubes, and 2 × sodium dodecyl sulphate buffer was added in a 1:1 ratio. Samples were boiled for 5 min and cooled on ice prior to Western blot analysis. Proteins (50 µg/sample for HSP70) were separated using 10% polyacrylamide gel electrophoresis. The proteins were transferred to polyvinylidene fluoride (PVDF) membranes using electrophoresis. Also, the PVDF membrane was blocked to membranes at room temperature by using skim-milk powder in TBST buffer for at least 1 h [35]. The HSP70 antibody (Enzo Life Sciences, Inc., New York, NY, USA) and β-actin antibody (Sigma Chemicals, Saint Louis, MO, USA) were added and incubated at 4 °C overnight. The membranes were washed three times with TBST (0.1% Tween and 2% 1 × Tris-buffered Hydrochloric acid) and incubated with anti-rabbit (mouse) immunoglobulin G horseradish peroxidase-conjugated secondary antibody for 1 h at room temperature (purchased from Beijing Zhong Shan Golden Bridge Biological Technology Co., Ltd., Beijing, China, dissolved in 5% non-fat dry milk and TBST at 1:5000 dilution). Then, the membranes were rinsed with TBST buffer 3 times for 10 min after incubation with a primary antibody and 5 times for 8 min after incubation with a secondary antibody. The enhanced chemiluminescence Western blotting kit (ECL-plus, Amersham Biosciences, Uppsala, Sweden) was used for blotting, the Gene Genome bioimaging system was used for imaging, and the GeneTools software (version 4.02 SPA) (Syngene, Frederick, MD, USA) was used for analysis.

### 2.10. Determination of the Antioxidant Capacity of Longissimus Dorsi Muscle

The concentrations of malondialdehyde (MDA) and hydrogen peroxide (H_2_O_2_), as well as the activities of superoxide dismutase (SOD) and catalase (CAT) were determined using commercially available kits (Nanjing Jiancheng Bioengineering Institute, Nanjing, China). The experiment was performed in triplicate.

### 2.11. Statistical Analysis

Data were reported as means with SD and were analysed using the SPSS 13.0 statistical software package (SPSS Inc., Chicago, IL, USA). The normality and constant variance for experimental data were tested using Levene’s test. Differences between means were determined using an unpaired *t*-test. Probability values < 0.05 indicated statistical significance.

## 3. Results

### 3.1. Determination the Activity of DAO in Plasma and Intestinal Morphology

**Figure 1:**
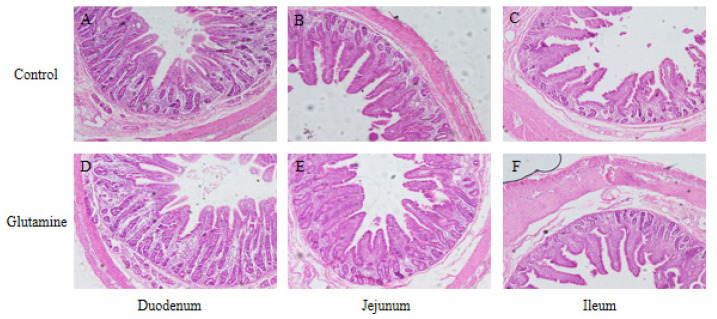
Intestinal villus morphology of the duodenum, jejunum, and ileum of piglets. The intestinal morphology of the duodenum, jejunum, and ileum in the Control group is shown in (**A**–**C**), respectively. The intestinal morphology of the duodenum, jejunum, and ileum of the Gln group is shown in (**D**–**F**), respectively. The morphology and structure of villi were complete and clear in outline.

The intestinal section morphology of the weaned piglets in the Control group and the Gln group is shown in Figure 1. Compared with the Control group, dietary Gln supplementation decreased (*p* < 0.05) DAO activity in plasma and increased VH, VH/CD, and VS in the small intestine of piglets (*p* < 0.05) (Figure 1 and Table 3).

### 3.2. The Contents of TP, RNA, and DNA in the Intestinal Mucosa of Piglets

Compared with the Control group, the dietary supplementation of Gln increased (*p* < 0.05) the content of TP, the RNA/DNA ratio, and the TP/DNA ratio in the mucosa of the jejunum and ileum, while decreasing the DNA content in the ileum mucosa (*p* < 0.05). There were no significant differences in the levels of these indexes in the duodenum (Table 4).

### 3.3. Concentrations of Amino Acids in the Jejunum and Ileum

Data on free amino acid concentrations in the jejunum and ileum are summarised in Table 5. Dietary Gln supplementation increased (*p* < 0.05) citrulline concentrations and decreased (*p* < 0.05) Gln and carnosine concentrations in the jejunal mucosa. In addition, the concentrations of citrulline, cystine, and β-alanine in the ileum mucosa were increased (*p* < 0.05), and the concentrations of Gln, β-aminoisobutyric acid, and 1-Methylhistidine in the ileum mucosa were decreased in the Gln group (*p* < 0.05).

### 3.4. Intestinal Gene Expression and Colon Bacterial Abundance

Data of the expression levels of genes in the duodenum, jejunum, and ileum are summarised in Table 6. Compared with the Control group, the dietary supplementation of Gln reduced (*p* < 0.05) the mRNA levels of *MX1*, *OAS1*, *IFIT1*, *IFIT2*, *IFIT3*, and *IFIT5* in the jejunum, and *IFIT1* and *MX1* in the ileum; and increased (*p* < 0.05) the mRNA levels of *GSTO2* and *IFN-β* in the duodenum, *GSTO2*, *IFN-α*, and *SLC7A6* in the jejunum, and *GSTO2*, *IFN-β*, *SLC6A19*, and *SLC7A7* in the ileum. In terms of colonic bacterial abundance, Gln supplementation piglets had a higher number of *Lactobacillus* genus than the Control group (*p* < 0.05) and lower numbers of *Enterobacterium* family, *Enterococcus* genus, and *Clostridium coccoides* than the Control group (*p* < 0.05) (Table 4).

### 3.5. Concentrations of Amino Acids in the Longissimus Dorsi Muscle

Data on the concentrations of free amino acid in the longissimus dorsi muscle are summarised in Table 7. Gln supplementation in diets increased (*p* < 0.05) the concentrations of citrulline, ornithine, arginine, and hydroxyproline, and decreased (*p* < 0.05) the concentration of Gln in the longissimus dorsi muscle.

### 3.6. TP, RNA, and DNA in the Longissimus Dorsi Muscle and Muscle Gene Expression

As shown in Table 8, Gln supplementation in diets not only increased (*p* < 0.05) the concentration of TP, DNA, and TP/DNA in the longissimus dorsi muscle, but also increased (*p* < 0.05) the mRNA level of *PepT1*.

### 3.7. Redox Status of Longissimus Dorsi Muscle

Data on the redox state of the longissimus dorsi muscle of piglets is summarized in Table 9. Gln supplementation in diets increased (*p* < 0.05) the activity of SOD. In addition, the concentrations of oxidative products, such as MDA and H_2_O_2_, were decreased (*p* < 0.05).

### 3.8. Effect of Gln Supplementation on the Abundance of the HSP70 Protein in the Jejunum Mucosa of Piglets

**Figure 2:**
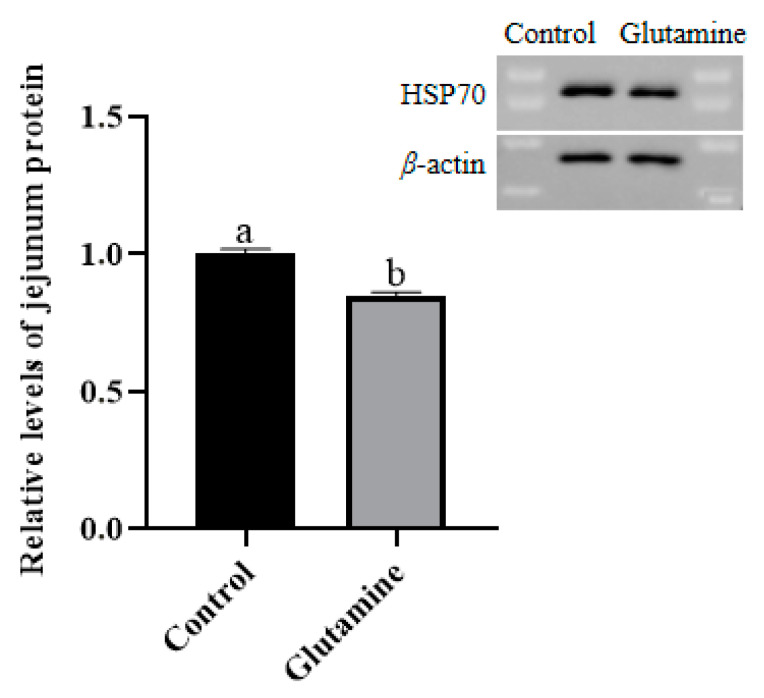
Effect of Gln supplementation on the abundance of the HSP70 protein in the jejunum mucosa of piglets. Data are mean ± SD, n = 3. ^a,b^ Mean values with different letters for same protein differ significantly (*p* < 0.05).

The expression level of HSP70 in the jejunal mucosa of piglets is shown in Figure 2. The expression level of HSP70 in the jejunal mucosa of the Gln group was significantly lower than that of the Control group.

## 4. Discussion

With increasing research into amino acids, there is growing recognition of the important impacts of amino acids on the health, growth, development, reproduction, and homeostasis of living organisms. In recent decades, biomedical and nutritional science have increasingly focused their attention on the beneficial effects of amino acids on animal intestinal health. Gln is widely recognised as a major metabolic fuel for the small intestine and serves as a substrate for intestinal cells to produce purines [9]. In addition, research has shown that Gln could stimulate enterocyte protein synthesis through the activation of the mTOR signalling pathway [8]. The aim of this study was to investigate the effects of Gln on improving intestinal function and promoting muscle growth. Our experimental results demonstrated that Gln increased VS, VW, and VH in the small intestine of piglets. Gln supplementation also enhanced mucosal TP levels as well as the RNA/DNA ratio and TP/DNA ratio in the jejunum and ileum [36]. Furthermore, we observed lower plasma DAO activity in piglets fed with Gln; also, DAO can be used to monitor mucosal injury severity [37]. Intriguingly, supplementation with Gln also led to increased levels of citrulline in both the jejunum and ileum, which possess protective effects on intestinal epithelial cells [38]. This increase in citrulline could potentially be attributed to referential enhancement by glutamine supplements within the intestine’s production capacity for citrulline itself [39]. It was also noted that the concentration of Gln decreased in both the jejunum and ileum; this result may be attributed to the fact that exogenous Gln promotes the proliferation of mucosal cells, which utilises Gln as an energy source, leading to the increased consumption and decreased content of Gln. These results suggest that Gln can enhance intestinal development and maintain its integrity.

Gln plays a role in regulating the redox status of the small intestine in piglets. As a precursor of glutathione synthesis [40], Gln protects the intestinal mucosa from peroxidation damage [41]. In this study, dietary supplementation with Gln was found to elevate the mRNA level of *GSTO2* in the small intestine of piglets. *GSTO2* can be directly reduced by glutathione or enzymatically by various thiol transferases and NADPH-dependent reductases [42]. Therefore, Gln supplementation improves the antioxidant capacity of the intestine in piglets. Furthermore, HSP70 is considered a marker for oxidative stress [43]; it serves as an important protective factor for intestinal epithelial cells against harmful substances and ulcer formation, and its expression promotes cell proliferation while inhibiting apoptosis and protein synthesis [44]. Notably, HSP70 is well known for its ability to protect organisms from heat-induced toxicity [45]. It also enhances cellular tolerance under conditions such as heat stress, oxidative stress, metal ion stress infection, and tumour growth through anti-apoptotic mechanisms and antioxidative properties. The decrease in HSP70 protein abundance observed in the jejunal mucosa supports the notion that dietary supplementation with Gln benefits intestinal function by reducing intestinal stress effects compared to Control group conditions.

The dietary supplementation of glutamine increased the mRNA levels of *IFN-α* in the jejunum and ileum and the mRNA levels of *IFN-β* in the duodenum and ileum, whereas it decreased interferon-stimulated genes (ISGs) [46], including *MX1*, *OAS1*, *IFIT1*, *IFIT2*, *IFIT3*, and *IFIT5*. Previous evidence has shown that virus infection can promote the expression of different ISGs in host cells [47,48,49]. In this study, the decrease in mRNA levels of some ISGs may be due to the fact that Gln can create a healthier environment within the intestinal tract.

In terms of intestinal nutrient transporters, dietary supplementation with Gln resulted in the increased expression of genes encoding amino acid transporters (*SLC6A19*, *SLC7A7*, and *SLC7A6*). *SLC6A19* is responsible for the transport of cationic amino acids across the apical membrane. Simultaneously, the main role of *SLC7A7* and *SLC7A6* is to not only transport neutral and cationic amino acids across the basolateral membrane enterocytes [50], but to also facilitate the absorption of peptides and amino acids [51]. Gln supplementation also elevated the mRNA levels of *PepT1* in the longissimus dorsi muscle which is elevated for piglet muscle growth [52]. *PepT1* belongs to the proton-dependent oligopeptide transporter family (POT). Many studies have confirmed its critical role in dipeptide, tripeptide, and pseudopeptide absorption within the intestinal tract [53]. Furthermore, the function of *PepT1* is associated with cellular networks regulating amino acid homeostasis, including the proteolysis of dietary proteins by proteases and peptidases, mTOR signalling, de novo protein synthesis, and the unfolded protein response [54]. As mentioned earlier, the dietary supplementation of Gln may enhance the intestinal absorption of water and amino acids by regulating the expression of epithelial aquaporin and amino acid transporters, thereby affecting the intestinal health of piglets.

Intestinal bacteria and amino acids are closely interconnected within the intestine. Their interaction significantly impacts host amino acid homeostasis and health as well as the efficiency of dietary amino acid supplementation [55]. The dietary supplementation of glutamine altered the piglet intestinal flora composition by reducing the abundance of *Clostridium coccoides*, the *Enterobacterium* family, and the *Enterococcus* genus, while increasing *Lactobacillus* abundance in the colon. The ratio between lactobacilli and intestinal bacteria can serve as an indicator of piglet intestinal health [33]. It has been suggested that effects of Gln on intestinal bacteria may be achieved by regulating specific enzyme activity [56], improving protein and energy balance [55], as well as enhancing the utilisation and metabolism of amino acids in the small intestine [6]. In summary, a 0.81% Gln dietary supplementation can maintain the intestinal health of piglets by improving gut microbiota composition.

One notable finding from this study is that Gln promotes muscle growth. The group supplemented with Gln exhibited increased concentrations of TP, RNA/DNA, and TP/DNA ratios in the longissimus dorsi muscle, indicating enhanced muscle synthesis capability [57]. The amino acid content in the longissimus dorsi muscle exhibited a significant decrease. Previous studies have demonstrated that the content of Gln in the longissimus dorsi muscle may significantly decrease during the growing phase of pigs due to its requirement for growth and development [52], which also indicates an increase in tissue protein synthesis [58]. Dietary supplementation with Gln resulted in an increase in the concentration of citrulline, ornithine, arginine, and hydroxyproline. Some studies of these amino acids have shown that citrulline can stimulate muscle protein synthesis by redistributing ATP consumption towards the process of muscle protein synthesis [59]; ornithine can inhibit the breakdown of proteins in skeletal muscle; and hydroxyproline constitutes a substantial proportion of total amino acids in collagen, which is an important component of muscle [60].

SOD and CAT are the key enzymes of the antioxidant system, playing crucial roles in scavenging free radicals and reducing oxidative damage. Under stress conditions, the activity of SOD and CAT in the serum decreased and the content of MDA increased [61]. He et al. [62] found that the supplementation of 1% Gln increased the activity of SOD but decreased the content of MDA. In this study, Gln supplementation improved the activity of SOD, but reduced the content of MDA, indicating that Gln may benefit from the growth of weaning pigs by enhancing antioxidant capacity and preventing peroxide damage to improve antioxidant capacity in the longissimus dorsi muscle.

## 5. Conclusions

These results suggest that Gln supplementation promotes the intestinal function of piglets by improving intestinal morphology, enhancing intestinal antioxidant capacity, increasing amino acid transporters and water channel transporters, and regulating intestinal flora. In addition, it also accelerates the growth of the longissimus dorsi muscle.

## Figures and Tables

**Table 1:** Ingredients and nutrient composition of the basal diet (air-dry basis).

Items	Content	Items	Content
Ingredient		Nutrient level	
Corn	38.4	DE (MJ/kg)	14.27
Soybean meal	16	CP	18.54
Flour	12	Lys	1.5
Whey powder (low protein)	8	Met	0.42
Soybean protein concentrate	5	Cys	0.29
Wheat middling	5	Thr	0.93
Fish meal	4.5	Trp	0.23
Glucose	3	Ca	0.75
CaHPO_4_	1.33	AP	0.49
Limestone	0.37	TP	0.68
NaCl	0.25	Na	0.31
Plant oil	3.95	CF	6.26
Premix *	1	NaCl	0.61
Lys	0.64		
Met	0.13		
Thr	0.21		
Choline	0.12		
Mildew preventive	0.1		

* The premix provided the following amounts of vitamins and trace minerals per kg of complete diet: Fe, 100 mg (FeSO_4_·H_2_O); Cu, 150 mg (CuSO_4_·5H_2_O); Mn, 40 mg (MnSO_4_·5H_2_O); Zn, 100 mg (ZnSO_4_·7H_2_O); I, 0.5 mg (KI); Se, 0.3 mg (Na_2_SeO_3_·5H_2_O); VA, 3.72 mg; VD_3_, 0.10 mg; DL-α-tocopheryl acetate, 26.7 mg; VK_3_, 4 mg; VB_1_, 6 mg; VB_2_, 12 mg; VB_6_, 6 mg; VB_12_, 0.05 mg; biotin, 0.2 mg; folic acid, 2 mg; niacin, 50 mg; and d-calcium pantothenate, 5 mg.

**Table 2:** The sequences of primers used in the present study.

Items	Primer Sequence (5′-3′)
Total bacteria count	(F) CGGTCCAGACTCCTACGGG
	(R) TTACCGCGGCTGCTGGCAC
*Enterococcus* genus	(F) CCCTTATTGTTAGTTGCCATCATT
	(R) ACTCGTTGTACTTCCCATTGT
*Enterobacterium* family	(F) CATTGACGTTACCCGCAGAAGAAGC
	(R) CTCTACGAGACTCAAGCTTGC
*Clostridium coccoides*	(F) AATGACGGTACCTGACTAA
	(R) CTTTGAGTTTCATTCTTGCGAA
*Lactobacillus* genus	(F) TCGCGTC(C/T)GGTGTGAAAG
	(R) CCACATCCAGC(A/G)TCCAC
Internal reference	(F) CAGAAATGGGAATGGAAAGTTG
	(R) CCATTGGTCAGGTCATTCAATACA
ASCT2	(F) GCCAGCAAGATTGTGGAGAT
	(R) GAGCTGGATGAGGTTCCAAA
GSTO2	(F) GCCTTGAGATGTGGGAGAGAA
	(R) AAGATGGTGTTCTGATAGCCAAGA
IFIT1	(F) GCTAAACCAAACACCGCAGAA
	(R) GGAACTCAATCTCCTCCAAGACC
IFIT2	(F) CAGAAGGCGGCAGAGAATG
	(R) ACACAGAGGCAGGCGAGATAG
IFIT3	(F) GCATTTTCCAGCCAGCATC
	(R) TCTGTTCCTTTCCTTTCCTTCCT
IFIT5	(F) CAGAAAATACAGCCATCCACCA
	(R) AGGGCACTTAAACTCTGCACATC
IFN-α	(F) ACTCCATCCTGGCTGTGAGGAAAT
	(R) ATCTCATGACTTCTGCCCTGACGA
IFN-β	(F) AGCAGATCTTCGGCATTCTC
	(R) GTCATCCATCTGCCCATCAA
MSTN	(F) GAAGTCAAGGTAACAGACACACCAA
	(R) GCAATAATCCAGTCCCATCCA
MX1	(F) AGTGCGGCTGTTTACCAAG
	(R) TTCACAAACCCTGGCAACTC
MX2	(F) CGCATTCTTTCACTCGCATC
	(R) CCTCAACCCACCAACTCACA
Myf5	(F) CCACGACTAACCCCAACCA
	(R) TTTTCCACCTGCTCCCTCA
OAS1	(F) TGGTGGTGGAGACACACACA
	(R) CCAACCAGAGACCCATCCA
OASL	(F) GGCACCCCTGTTTTCCTCT
	(R) AGCACCGCTTTTGGATGG
PepT1	(F) ATTCTCAGGCTCCTTCCAACA
	(R) GCAACCCCGCAAACAGA
SLC7A6	(F) CTGCCGCCTGCATGTGT
	(R) TGTGCCCCACTTGACATAGG
SLC7A7	(F) TTTGGTTCCCAAGGTTGCA
	(R) GCAGCTTCCTGGCATTGC
SLC6A19	(F) CGAGTACCCGTACCTGATGGA
	(R) TGCGTAGAAGGGCGAAGAA

**Table 3:** Effects of dietary supplementation with Gln on plasma DAO and intestinal morphology in piglets.

Items	Control Group	Gln Group	*p*-Value
Plasma			
DAO (mmol/L)	15.36 ± 2.49 ^a^	9.38 ± 3.54 ^b^	0.002
Duodenum			
Villus height (μm)	282 ± 24 ^b^	349 ± 13 ^a^	<0.01
Crypt depth (μm)	119 ± 7	121 ± 7	0.574
Villus height/crypt depth	2.45 ± 0.16 ^b^	2.89 ± 0.23 ^a^	<0.01
Villus width (μm)	140 ± 8 ^b^	161 ± 5 ^a^	<0.01
Villus surface area (μm^2^)	32,431 ± 3438 ^b^	44,744 ± 3273 ^a^	<0.01
Jejunum			
Villus height (μm)	260 ± 21 ^b^	311 ± 31 ^a^	0.002
Crypt depth (μm)	99 ± 7	103 ± 16	0.564
Villus height/crypt depth	2.60 ± 0.14 ^b^	3.06 ± 0.36 ^a^	0.006
Villus width (μm)	104 ± 5 ^b^	124 ± 8 ^a^	<0.01
Villus surface area (μm^2^)	20,481 ± 3519 ^b^	26,809 ± 3987 ^a^	0.005
Ileum			
Villus height (μm)	231 ± 16 ^b^	290 ± 22 ^a^	<0.01
Crypt depth (μm)	110 ± 12	114 ± 16	0.631
Villus height/crypt depth	2.18 ± 0.10 ^b^	2.59 ± 0.30 ^a^	0.002
Villus width (μm)	106 ± 6 ^b^	120 ± 10 ^a^	0.005
Villus surface area (μm^2^)	18,462 ± 1742 ^b^	25,847 ± 3745 ^a^	<0.01

Data are mean ± SD, n = 8. ^a,b^ Means within rows with different superscripts differ signficantly (*p* < 0.05).

**Table 4:** Effects of dietary supplementation with Gln on TP, RNA, and DNA in the intestinal mucosa in piglets.

Items	Control Group	Gln Group	*p*-Value
Duodenum			
TP (mg/g)	67.20 ± 7.10	66.00 ± 3.90	0.694
RNA (mg/g)	2.51 ± 0.32	2.58 ± 0.34	0.522
DNA (mg/g)	0.36 ± 0.07	0.40 ± 0.07	0.234
RNA/DNA	7.27 ± 1.66	6.91 ± 1.84	0.256
TP/DNA	196.64 ± 45.10	175.37 ± 35.97	0.716
Jejunum			
TP (mg/g)	51.50 ± 7.60 ^b^	62.70 ± 6.10 ^a^	0.006
RNA (mg/g)	1.70 ± 0.33	1.75 ± 0.24	0.744
DNA (mg/g)	0.21 ± 0.02	0.19 ± 0.02	0.162
RNA/DNA	7.79 ± 1.04 ^b^	8.80 ± 0.46 ^a^	<0.01
TP/DNA	255.12 ± 61.68 ^b^	319.59 ± 52.72 ^a^	0.001
Ileum			
TP (mg/g)	31.90 ± 6.70 ^b^	58.90 ± 7.30 ^a^	<0.01
RNA (mg/g)	2.24 ± 0.36	2.22 ± 0.30	0.709
DNA (mg/g)	0.18 ± 0.026 ^a^	0.14 ± 0.01 ^b^	0.001
RNA/DNA	11.49 ± 2.67 ^b^	15.25 ± 2.85 ^a^	0.020
TP/DNA	166.67 ± 30.90 ^b^	402.96 ± 61.66 ^a^	<0.01

Data are mean ± SD, n = 8. ^a,b^ Means within rows with different superscripts differ significantly (*p* < 0.05).

**Table 5:** Effects of dietary supplementation with Gln on the concentrations of amino acids in the jejunum and ileum of piglets.

Amino Acids (μg/g)	Control Group	Gln Group	*p*-Value
Jejunum			
1-Methylhistidine	4.081 ± 1.896	4.103 ± 2.893	0.986
Arginine	216.335 ± 61.647	210.695 ± 66.219	0.863
Carnosine	6.582 ± 1.502 ^a^	4.55 ± 1.153 ^b^	0.009
Citrulline	94.349 ± 26.045 ^b^	160.174 ± 47.633 ^a^	0.004
Cystine	2.489 ± 0.649	4.731 ± 3.406	0.089
Glutamine	833.294 ± 133.851 ^a^	638.748 ± 108.339 ^b^	0.006
Hydroxyproline	112.917 ± 5.053	110.441 ± 15.041	0.666
Ornithine	45.68 ± 7.7	43.54 ± 5.49	0.533
β-Alanine	60.883 ± 8	74.941 ± 21.413	0.104
β-Aminoisobutyric acid	17.703 ± 6.117	20.05 ± 23.795	0.791
Ileum			
1-Methylhistidine	2.956 ± 0.463 ^a^	1.869 ± 0.566 ^b^	0.001
Arginine	187.077 ± 19.474	184.844 ± 39.183	0.887
Carnosine	7.565 ± 2.58	7.607 ± 2.256	0.973
Citrulline	62.951 ± 10.771 ^b^	87.347 ± 22.556 ^a^	0.015
Cystine	2.939 ± 0.613	3.598 ± 1.069	0.152
Glutamine	983.157 ± 186.976 ^a^	679.397 ± 140.461 ^b^	0.003
Hydroxyproline	167.301 ± 17.16 ^a^	142.792 ± 7.786 ^b^	0.002
Ornithine	110.647 ± 18.887	120.151 ± 52.105	0.635
β-Alanine	67.41 ± 16.363 ^b^	86.473 ± 17.62 ^a^	0.042
β-Aminoisobutyric acid	2.676 ± 0.613 ^a^	1.425 ± 0.133 ^b^	<0.01

Data are mean ± SD, n = 8. ^a,b^ Means within rows with different superscripts differ significantly (*p* < 0.05).

**Table 6:** Effects of dietary supplementation with Gln on the expression levels of genes in the duodenum, jejunum, and ileum and bacterial genes in the colon in piglets.

Items	Control Group	Gln Group	*p*-Value
Duodenum			
GSTO2	1.000 ± 0.213 ^b^	3.749 ± 0.913 ^a^	<0.01
IFN-β	1.000 ± 0.119 ^b^	2.684 ± 0.649 ^a^	<0.01
Jejunum			
GSTO2	1.000 ± 0.257	1.254 ± 0.274	0.077
IFIT1	1.000 ± 0.281 ^a^	0.532 ± 0.138 ^b^	0.001
IFIT2	1.000 ± 0.248 ^a^	0.613 ± 0.163 ^b^	0.002
IFIT3	1.000 ± 0.163 ^a^	0.661 ± 0.139 ^b^	0.001
IFIT5	1.000 ± 0.119 ^a^	0.659 ± 0.158 ^b^	<0.01
IFN-α	1.000 ± 0.245 ^b^	1.286 ± 0.272 ^a^	0.044
MX1	1.000 ± 0.143 ^a^	0.464 ± 0.106 ^b^	<0.01
MX2	1.000 ± 0.265 ^a^	0.600 ± 0.155 ^b^	0.002
OAS1	1.000 ± 0.272 ^a^	0.670 ± 0.175 ^b^	0.012
OASL	1.000 ± 0.281 ^a^	0.446 ± 0.111 ^b^	<0.01
SLC7A6	1.000 ± 0.154 ^b^	1.227 ± 0.236 ^a^	0.039
Ileum			
GSTO2	1.000 ± 0.118 ^b^	2.005 ± 0.399 ^a^	<0.01
IFIT1	1.000 ± 0.224 ^a^	0.607 ± 0.135 ^b^	0.001
IFN-α	1.000 ± 0.169 ^b^	1.321 ± 0.275 ^a^	0.014
IFN-β	1.000 ± 0.101 ^b^	1.392 ± 0.293 ^a^	0.003
MX1	1.000 ± 0.161 ^a^	0.712 ± 0.148 ^b^	0.002
SLC7A7	1.000 ± 0.235 ^b^	1.291 ± 0.251 ^a^	0.031
*Colon bacteria*			
*Clostridium coccoides*	1.000 ± 0.154 ^a^	0.335 ± 0.059 ^b^	<0.01
*Enterobacterium* family	1.000 ± 0.136 ^a^	0.354 ± 0.044 ^b^	<0.01
*Enterococcus* genus	1.000 ± 0.246 ^a^	0.728 ± 0.181 ^b^	0.024
*Lactobacillus* genus	1.000 ± 0.242 ^b^	2.450 ± 0.449 ^a^	<0.01

Data are mean ± SD, n = 8. The expression level of the Control group was regarded as 1. ^a,b^ Means within rows with different superscripts differ significantly (*p* < 0.05).

**Table 7:** Effects of dietary supplementation with Gln on the concentrations of amino acids in the longissimus dorsi muscle of piglets.

Amino Acids	Control Group	Gln Group	*p*-Value
1-Methylhistidine (μg/g)	3.955 ± 1.209	3.743 ± 1.437	0.755
Arginine (μg/g)	56.966 ± 8.866 ^b^	107.416 ± 52.704 ^a^	0.018
Carnosine (ng/g)	13.65 ± 0.916	14.044 ± 1.411	0.519
Citrulline (μg/g)	57.191 ± 11.348 ^b^	82.69 ± 14.84 ^a^	0.002
Glutamine (ng/g)	2.075 ± 0.328 ^a^	1.629 ± 0.233 ^b^	0.007
Hydroxyproline (μg/g)	94.618 ± 12.03 ^b^	124.028 ± 23.401 ^a^	0.007
Ornithine (μg/g)	155.918 ± 33.51 ^b^	222.05 ± 59.788 ^a^	0.016
β-Alanine (ng/g)	0.962 ± 0.107	0.857 ± 0.225	0.254
β-Aminoisobutyric acid (μg/g)	14.926 ± 5.312	16.039 ± 3.385	0.625

Data are mean ± SD, n = 8. ^a,b^ Means within rows with different superscripts differ significantly (*p* < 0.05).

**Table 8:** Effects of dietary supplementation with Gln on TP, RNA, and DNA and the expression levels of genes in the longissimus dorsi muscle in piglets.

Items	Control Group	Gln Group	*p*-Value
TP (mg/g)	29.1 ± 2.2 ^b^	37.4 ± 5.1 ^a^	0.001
RNA (mg/g)	0.060 ± 0.017 ^a^	0.035 ± 0.007 ^b^	<0.01
DNA (mg/g)	0.336 ± 0.021 ^b^	0.386 ± 0.049 ^a^	0.003
RNA/DNA	0.170 ± 0.042 ^a^	0.091 ± 0.020 ^b^	<0.01
TP/DNA	87.771 ± 5.219 ^b^	100.078 ± 11.917 ^a^	<0.01
Genes			
ASCT2	1.000 ± 0.212	0.847 ± 0.179	0.140
SLC6A19	1.000 ± 0.230	1.102 ± 0.185	0.343
MSTN	1.000 ± 0.202	0.964 ± 0.217	0.738
Myf5	1.000 ± 0.164	1.087 ± 0.187	0.339
PepT1	1.00 ± 0.176 ^b^	1.569 ± 0.408 ^a^	0.003
SLC7A7	1.00 ± 0.189	0.878 ± 0.197	0.228
SLC7A6	1.000 ± 0.250	1.065 ± 0.249	0.611

Data are mean ± SD, n = 8. The expression level of the Control group was regarded as 1. ^a,b^ Means within rows with different superscripts differ significantly (*p* < 0.05).

**Table 9:** Effects of dietary supplementation with Gln on antioxidant capacity in the longissimus dorsi muscle of piglets.

Items	Control Group	Gln Group	*p*-Value
CAT (U/mg protein)	1.11 ± 0.33	1.09 ± 0.30	0.910
MDA (nmol/mg protein)	0.65 ± 0.15 ^a^	0.41 ± 0.20 ^b^	0.020
H_2_O_2_ (μmol/mg protein)	1.59 ± 0.53 ^a^	1.11 ± 0.25 ^b^	0.009
SOD (U/mg protein)	73.1 ± 12.03 ^b^	83.8 ± 7.11 ^a^	0.048

Data are mean ± SD, n = 8. ^a,b^ Means within rows with different superscripts differ significantly (*p* < 0.05).

## Data Availability

Data will be made available upon request.

## Abbreviations

ASCT2	Sodium-dependent neutral amino acid transporter 2
CAT	Catalase
DAO	Diamine oxidase
Gln	l-glutamine
GSTO2	Glutathione-S-transferase omega-2
H_2_O_2_	Hydrogen peroxide
HSP70	Heat shock protein-70
IFIT	Interferon induced protein with tetratricopeptide repeats
ISGs	Interferon stimulated genes
MDA	Malondialdehyde
MSTN	Myostatin
mTOR	Mammalian target of rapamycin
MX1	MX dynamin Like GTPase 1
MX2	MX dynamin like GTPase 2
Myf5	Myogenic Factor 5
OAS1	2′-5′-Oligoadenylate Synthetase 1
OASL	2′-5′-Oligoadenylate Synthetase Like
PepT1	Peptide transporter 1
SLC7A6	l-type amino acid transporter 2
SLC7A7	l-type amino acid transporter 1
SLC6A19	Sodium-independent amino acid transporter
SOD	Superoxide dismutase
TP	Total protein
VH	Villus height
VH/CD	Villus height/crypt depth ratio
VS	Villus surface area
VW	Villus width
